# In-stent restenosis of drug-eluting stents: clinical presentation and outcomes in a real-world scenario

**DOI:** 10.1186/s43044-019-0025-z

**Published:** 2019-11-27

**Authors:** Ganesh Paramasivam, Tom Devasia, Shabeer Ubaid, Ashwitha Shetty, Krishnananda Nayak, Umesh Pai, Mugula Sudhakar Rao

**Affiliations:** 1 Department of Cardiology, Kasturba Medical College, Manipal, Manipal Academy of Higher Education, Manipal, Karnataka 576104 India; 2 Department of Cardiovascular Technology, School of Allied Health Sciences, Manipal Academy of Higher Education, Manipal, Karnataka 576104 India

**Keywords:** In-stent restenosis, Drug-eluting stent, Percutaneous coronary intervention

## Abstract

**Background:**

Drug-eluting stents (DES) have substantially reduced the incidence of coronary in-stent restenosis (ISR), but the problem persists. Clinical presentation and outcomes of DES-ISR in a real-world scenario remains underreported.

**Results:**

In this retrospective study, we examined medical records of 191 consecutive patients with DES-ISR (210 ISR lesions) hospitalized between January 2013 and December 2017. ISR clinical presentation was classified as acute coronary syndrome (ACS) or non-ACS. Clinical, angiographic features and 1-year outcomes [composite of death, myocardial infarction (MI) and repeat-target lesion revascularization] for these two groups were compared.

The mean age of study population was 61 ± 10 years and 81.2% were males. ACS was the dominant clinical presentation mode occurring in 118 (61.8%) patients. MI was seen in 66 (34.6%) patients. Female gender (odds ratio, 2.71; 95% confidence interval [CI], 1.13–6.52; *P* = 0.026) and chronic kidney disease (odds ratio, 3.85; 95% CI, 1.05–14.20; *P* = 0.043) correlated significantly with ACS ISR presentation. A majority [104 (54.5%)] of patients underwent percutaneous coronary intervention (PCI), of whom 72 (69.2%) received a new DES. The rest either underwent CABG (26.2%) or received medical therapy (19.4%). Patients presenting with ACS had a significantly worse clinical outcome at 1-year follow-up (ACS versus non-ACS presentation: hazard ratio [HR], 2.66; 95% CI, 1.09–6.50; *P* = 0.032).

**Conclusions:**

DES-ISR presents most commonly as ACS. Female gender and chronic kidney disease seem to be associated with ACS presentation. ACS presentation of ISR is associated with worse 1-year outcomes. Early identification of those with ACS risk and closer follow-up may improve outcomes.

## Background

Restenosis is the Achilles’ heel of coronary intervention [1]. Bare-metal stents (BMS) reduced the restenosis rates seen with balloon angioplasty predominantly by mitigating the effects of elastic recoil and negative remodelling [2, 3]. However, the vascular response to injury in the form of neointimal hyperplasia offset the aforementioned benefits and led to in-stent restenosis (ISR) [4]. Introduction of DES, which was designed to minimize this problem, resulted in two important changes. First, the incidence of ISR reduced to 5–10% compared to BMS era where up to a third of the patients presented with restenosis [5–9]. Second, focal angiographic ISR pattern became more common compared to BMS ISR which presented commonly with a diffuse pattern [10]. Considering the increasing DES usage in the present era worldwide, even this lower ISR incidence with DES still accounts for a large number of cases yearly.

Initial studies of DES ISR focused on the occurrence of angiographic ISR, but a significant proportion of ISR cases remain asymptomatic and thus may not be relevant clinically. Clinical ISR (presence of symptoms or objective evidence of myocardial ischemia attributable to ISR) presents a therapeutic challenge and carries prognostic implications. Only a few studies have explored the relationship between the types of ISR clinical presentation and outcomes mostly following a retrospective design. Most such studies have included only those patients who underwent percutaneous coronary intervention (PCI) for culprit ISR lesion [11, 12]. Since in the real world scenario patients may be treated with medical therapy, percutaneous or surgical revascularization, these studies possess an inherent selection bias and may not be representative of the entire clinical ISR population. We set out to study clinical ISR of DES in the real-world scenario, focusing on the presentation mode and its impact on clinical outcomes.

## Methods

### Study population and design

**Fig. 1:**
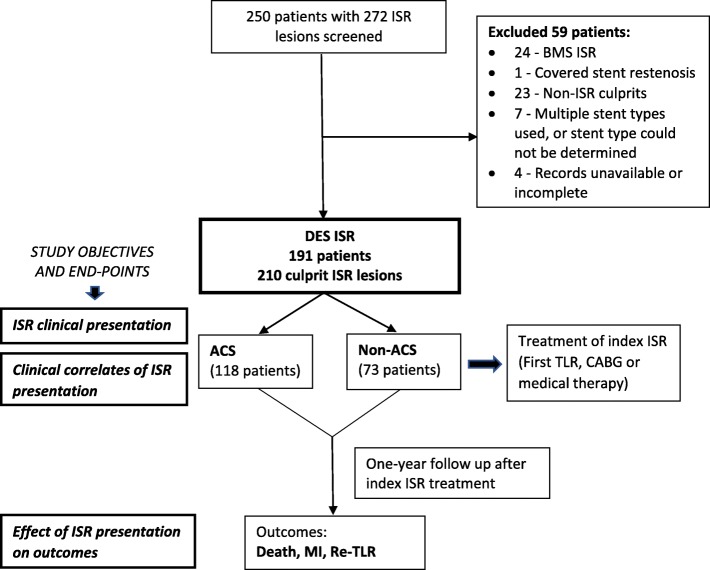
Study flow chart. ACS: acute coronary syndrome; BMS: bare-metal stent; CABG: coronary artery bypass grafting; DES: drug-eluting stent; ISR: in-stent restenosis, MI: myocardial infarction; TLR: target-lesion revascularization

This single-centre, retrospective cohort study included consecutive patients with clinical culprit ISR lesions of drug-eluting stents (DES) diagnosed between January 2013 and December 2017 at a tertiary care hospital in South India (Fig. 1). Approval for the study protocol was obtained from the institutional ethics committee.

Medical records of these patients were examined. Clinical and demographic characteristics including risk factors for atherosclerotic cardiovascular disease (ASCVD) and investigations including results of blood tests, electrocardiography and echocardiography tests were recorded. Angiographic images were reviewed by two independent cardiologists who confirmed the presence of ISR and also determined the ISR type (as described by Mehran et al.) [13]. Details of treatment given for the culprit ISR lesion, including interventional procedures, were noted. Also, details of initial PCI (prior to ISR diagnosis), when available, including the type of DES used were recorded.

### Study definitions

In-stent restenosis was defined angiographically as the presence of > 50% diameter stenosis at the stent site or at its edges (adjacent 5 mm segments) [14]. Clinical ISR was diagnosed when symptoms and/or inducible ischemia (on stress tests) were present and were attributable to the ISR lesion.

Index hospitalization was the hospitalization event during which ISR was first diagnosed. Clinical presentation during this hospitalization was classified into ACS (acute coronary syndrome) which included patients with unstable angina (UA) and myocardial infarction (MI), and non-ACS which included patients diagnosed with either stable angina or silent ischemia.

Diagnosis of MI was based on the universal definition and was categorized into STEMI (ST-elevation myocardial infarction) and NSTEMI (non-ST elevation myocardial infarction) [15]. Typical chest discomfort brought upon by physical exertion and relieved by rest and/or nitrates was diagnosed as stable angina. Unstable angina was defined as a recent onset or worsening of typical chest pain, chest pain lasting > 20 min and/or occurring at rest with or without ST-segment changes on electrocardiography and without an increase in the blood levels of cardiac biomarkers. Silent ischemia was defined as ischemia identified on stress tests (treadmill exercise test or dobutamine echocardiography) in the absence of symptoms [16].

The presence of chronic kidney disease was established using the definitions provided in the Kidney Disease: Improving Global Outcomes (KDIGO) 2012 guidelines [17]. Stent thrombosis was diagnosed using the criteria suggested by the Academic Research Consortium as definite or probable [18].

### Outcome definitions

The primary outcome was a composite outcome which included major adverse cardiac events (MACE) and mortality from all causes following index hospitalization. MACE included both repeat MI and repeat TLR (target-lesion revascularization) which occurred during the 1-year follow-up period. MACE did not include the revascularization procedure carried out for index ISR lesion or the MI event which led to index hospitalization. All deaths were assumed to be cardiac unless another clearly documented cause was available.

### Follow-up

Follow-up data for a duration of 1 year following the index hospitalization was obtained from patients’ hospital records to determine the occurrence of one or more of the outcome events defined above. Telephonic contact was used to collect follow-up data for patients in whom such data was not available from hospital/medical records. Two independent cardiologists who were blinded to the study objectives adjudicated all the outcome events.

### Study objectives

The main objective was to study the clinical presentation and its impact on the occurrence of MACE in patients with ISR in DES. The secondary objective was to determine the clinical correlates for ACS as the presentation mode for DES-ISR compared to non-ACS (Fig. 1).

### Statistical analysis

Continuous variables were described using mean and standard deviation. Frequencies (%) were used for categorical variables. Chi-square test or Fisher’s exact test was used for categorical variables and *t* test for continuous variables to compare groups with ACS and non-ACS presentations. Logistic regression analysis (univariate and multivariate) was used to test correlates for ACS as ISR presentation. Survival (time-to-event) analysis for adverse events according to ISR presentation mode was carried out using the Kaplan-Meier method and the log-rank test. Cox regression analysis was used to calculate the hazard ratio for the effect of clinical presentation mode on outcomes. *P* value of < 0.05 was taken as an indicator of statistical significance. Statistical analysis was carried out using SPSS software (version 16.0).

## Results

### Clinical and angiographic characteristics

This study included 191 patients with 210 culprit ISR lesions. Mean (± SD) age of the patients was 61 (± 10) years and 155 (81.2%) were males. Patient characteristics, stratified according to clinical presentation at the time of index hospitalization, are represented in Table 1. ACS was the dominant clinical presentation mode, occurring in 118 (61.8%) patients. Among patients presenting with ACS, 52 (44%) had UA, 52 (44%) had NSTEMI and 14 (12%) had STEMI. Among the 73 (38.2%) patients who presented as non-ACS, 63 (86.3%) had stable angina and the remaining 10 (13.7%) had silent ischemia which was diagnosed during stress testing. ACS cohort had a significantly higher proportion of women and patients with CCF and chronic kidney disease.

**Table 1:** Patient characteristics at first clinical ISR presentation

Parameter	Total (*n* = 191)	ACS cohort (*n* = 118)	Non-ACS cohort (*n* = 73)	*P* value
Demographics				
Age	61 ± 10	62 ± 10	59 ± 10	0.074
Men	155 (81.2%)	90 (76.3%)	65 (89.0%)	0.036
BMI	23.5 ± 3.5	23.7 ± 3.9	23.1 ± 2.7	0.479
Clinical characteristics				
Diabetes	109 (57.1%)	68 (57.6%)	41 (56.2%)	0.881
Hypertension^ƚ^	104 (54.5%)	66 (55.9%)	38 (52.1%)	0.655
Chronic kidney disease	21 (11.0%)	18 (15.3%)	3 (4.1%)	0.017
Acute kidney injury	32 (16.8%)	24 (20.3%)	8 (11.0%)	0.112
Dyslipidemia*	98 (67.1%)	55 (63.2%)	43 (72.9%)	0.282
Current tobacco use	39 (20.4%)	22 (18.6%)	17 (23.3%)	0.464
CCF	34 (17.8%)	30 (25.4%)	4 (5.5%)	< 0.001
NYHA 3,4	20 (10.5%)	14 (11.9%)	6 (8.2%)	0.476
LVEF	53 ± 11	51 ± 10	54 ± 11	0.076
Previous MI	100 (52.4%)	65 (55.1%)	35 (47.9%)	0.373
Previous CABG	14 (7.3%)	9 (7.6%)	5 (6.8%)	1.000
Statin therapy	163 (85.3%)	99 (83.9%)	64 (87.7%)	0.533
Lipid profile* (mg/dL)				
Total cholesterol	145 ± 42	146 ± 45	144 ± 37	0.785
LDL	80 ± 35	80 ± 38	79 ± 31	0.896
HDL	39 ± 12	41 ± 13	38 ± 10	0.126
Triglycerides	130 ± 71	124 ± 67	140 ± 76	0.166

*ACS* acute coronary syndrome, *BMI* body mass index, *CABG* coronary artery bypass grafting, *CCF* congestive cardiac failure, *LVEF* left ventricular ejection fraction, *LDL* low-density lipoprotein, *HDL* high-density lipoprotein, *MI* myocardial infarction, *NYHA* New York Heart Association

*Dyslipidemia defined as total cholesterol > 250 mg/dL, LDL cholesterol > 130 mg/dL, HDL cholesterol < 40 mg/dL (< 50 mg/dL for women) in the fasting state. Data available for 146 patients

^ƚ^Blood pressure > 140/90 mmHg or the use of antihypertensive therapy

Table 2 shows the angiographic characteristics, treatment strategy and characteristics of interventional procedures. For patients who underwent PCI for culprit ISR lesions, the details of the type of intervention including the type of stents and adjunctive devices used are shown. Focal ISR lesions were more common than all other ISR lesion types combined (63.8% vs. 36.2%). Type 1C Mehran type was the most common type of ISR lesion found in both ACS and non-ACS groups (34.1% and 35.8% respectively). There was no difference between the type of ISR lesions found in ACS and non-ACS groups (*P* = 0.961). Both groups were similar with respect to disease burden, vessels affected by ISR, ISR location and treatment received. PCI was the most common treatment modality in both groups, and more than two thirds of those patients received a new DES.

**Table 2:** Angiographic characteristics and treatment characteristics at first clinical ISR presentation

Parameter	Total	ACS cohort	Non-ACS cohort	*P* value
ISR characteristics	(*n* = 210)	(*n* = 129)	(*n* = 81)	
ISR type				0.961
I. Focal	134 (63.8%)	84 (65.1%)	50 (61.7%)	
II. Diffuse	23 (11.0%)	14 (10.9%)	9 (11.1%)	
III. Proliferative	10 (4.7%)	6 (4.7%)	4 (4.9%)	
IV. Complete	43 (20.5%)	25 (19.4%)	18 (22.2%)	
ISR vessel				0.203
Left anterior descending	112 (53.3%)	62 (48.1%)	50 (61.7%)	
Left circumflex artery	49 (23.3%)	33 (25.6%)	16 (19.8%)	
Right coronary artery	47 (22.4%)	32 (24.8%)	15 (18.5%)	
Left main	2 (1.0%)	2 (1.6%)	0 (0.0%)	
Proximal ISR location	113 (53.8%)	68 (52.7%)	45 (55.6%)	0.776
Disease burden	(*n* = 191)	(*n* = 118)	(*n* = 73)	0.715
Single-vessel disease	72 (37.7%)	42 (35.6%)	30 (41.1%)	
Double-vessel disease	67 (35.1%)	42 (35.6%)	25 (34.2%)	
Triple-vessel disease	52 (27.2%)	34 (28.8%)	18 (24.7%)	
Treatment plan	(*n* = 191)	(*n* = 118)	(*n* = 73)	0.620
Medical therapy	37 (19.4%)	24 (20.3%)	13 (17.8%)	
CABG	50 (26.2%)	33 (28.0%)	17 (23.3%)	
PCI	104 (54.5%)	61 (51.7%)	43 (58.9%)	
Details of PCI				
Procedural success	101 (97.1%)	59 (96.7%)	42 (97.7%)	1.000
PCI type				0.082
POBA	21 (20.2%)	16 (26.2%)	5 (11.6%)	
DCB	11 (10.6%)	4 (6.6%)	7 (16.3%)	
New DES	72 (69.2%)	41 (67.2%)	31 (72.1%)	
No. of stents	1.14 ± 0.35	1.16 ± 0.37	1.12 ± 0.33	0.608
Stent length	29.7 ± 11.0	32.0 ± 12.9	26.4 ± 6.4	0.045
Stent diameter	3.03 ± 0.40	3.00 ± 0.36	3.08 ± 0.45	0.451
Adjunct Devices				
Rotablation	2 (1.9%)	2 (3.3%)	0 (0.0%)	0.342
Cutting or NC balloon	24 (23.1%)	13 (21.3%)	11 (25.6%)	0.390
IVUS guidance	25 (24.0%)	13 (21.3%)	12 (27.9%)	0.292

*ACS* acute coronary syndrome, *CABG* coronary artery bypass grafting, *DCB* drug-coated balloon, *DES* drug-eluting stent, *IVUS* intravascular ultrasound, *ISR* in-stent restenosis, *NC* non-compliant, *PCI* percutaneous coronary intervention, *POBA* plain old balloon angioplasty

### Clinical correlates for ACS as ISR presentation

We analysed the clinical correlates for ACS as ISR presentation (Table 3). Female gender (odds ratio, 2.71; 95% CI, 1.13–6.52; *P* = 0.026) and chronic kidney disease (odds ratio, 3.85; 95% CI, 1.05–14.20; *P* = 0.043) correlated with ACS presentation. Patients with ACS were more likely to have CCF (odds ratio, 4.98; 95% CI, 1.63–15.26; *P* = 0.005). Age, body mass index, diabetes mellitus, hypertension, dyslipidemia, tobacco use, previous MI or previous CABG did not correlate with ACS ISR presentation. Lesion-related characteristics like proximal ISR location or involvement of the left anterior descending artery also did not correlate with an ACS presentation.

**Table 3:** Correlates of ACS ISR presentation compared to non-ACS presentation

Variables	Univariate analysis				Multivariate analysis			
	OR	Lower 95% CI	Upper 95% CI	*P* value	OR	Lower 95% CI	Upper 95% CI	*P* value
Patient-related								
Age	1.03	0.99	1.06	0.076	1.01	0.98	1.05	0.449
Female gender	2.53	1.08	5.90	0.032	2.71	1.13	6.52	0.026
BMI	1.04	0.93	1.17	0.476	–	–	–	–
Diabetes	1.06	0.59	1.91	0.843	–	–	–	–
Hypertension	1.17	0.65	2.10	0.601	–	–	–	–
Current tobacco use	0.76	0.37	1.54	0.440	–	–	–	–
Dyslipidemia	1.56	0.76	3.21	0.224	–	–	–	–
Chronic kidney disease	4.20	1.19	14.80	0.026	3.85	1.05	14.20	0.043
CCF	5.88	1.98	17.49	0.001	4.98	1.63	15.26	0.005
Previous MI	1.33	0.74	2.39	0.338	–	–	–	–
Previous CABG	1.12	0.36	3.49	0.841	–	–	–	–
Statin therapy	0.73	0.31	1.72	0.475				
Lesion-related								
LAD involvement	0.71	0.39	1.28	0.248	–	–	–	–
Proximal ISR location	0.88	0.49	1.58	0.656	–	–	–	–

*ACS* acute coronary syndrome, *BMI* body mass index, *CABG* coronary artery bypass grafting, *CCF* congestive cardiac failure, *CI* confidence interval, *ISR* in-stent restenosis, *LAD* left anterior descending, *LVEF* left ventricular ejection fraction, *MI* myocardial infarction, *OR* odds ratio

Angiographic and procedural characteristics of the initial PCI procedure can influence the development of ISR and potentially affect its clinical presentation. Such data was available in only 106 study patients as many of these patients presented for the first time to our hospital with ISR and/or medical records of initial PCI was unavailable. We have compared these variables among groups currently presenting with or without ACS (Table 4). These characteristics were not significantly associated with ACS ISR presentation.

**Table 4:** Angiographic and PCI characteristics during initial PCI

Parameter	Total (*n* = 106)	ACS cohort (*n* = 68)	Non-ACS cohort (*n* = 38)	*P* value
Lesion type				0.427
A	51 (48.1%)	29 (42.7%)	22 (57.9%)	
B	28 (26.4%)	19 (27.9%)	9 (23.7%)	
C	27 (25.5%)	20 (29.4%)	7 (18.4%)	
Calcification	14 (13.2%)	7 (10.3%)	7 (18.4%)	0.358
Disease burden				0.627
Single-vessel disease	55 (51.9%)	38 (55.9%)	17 (44.7%)	
Double-vessel disease	33 (31.1%)	18 (26.5%)	15 (39.5%)	
Triple-vessel disease	18 (17.0%)	12 (17.6%)	6 (15.8%)	
Details of PCI				
No. of stents	1.3 ± 0.6	1.3 ± 0.6	1.3 ± 0.6	0.923
Stent length	27.0 ± 8.7	28.2 ± 8.7	24.6 ± 8.6	0.124
Stent diameter	2.92 ± 0.34	2.93 ± 0.36	2.90 ± 0.31	0.712
Pre-dilation	63 (59.4%)	43 (63.2%)	20 (52.6%)	0.541
Post-dilation	43 (40.5%)	27 (39.7%)	16 (42.1%)	0.944
DES type				0.683
Paclitaxel	3 (2.8%)	3 (4.4%)	–	
Sirolimus	70 (66.1%)	46 (67.6%)	24 (63.2%)	
Everolimus	21 (19.8%)	12 (17.7%)	9 (23.7%)	
Zotarolimus	12 (11.3%)	7 (10.3%)	5 (13.1%)	
Time to ISR	26.8 ± 25	24.5 ± 24	31.9 ± 30.1	0.481

*ACS* acute coronary syndrome, *DES* drug-eluting stent, *PCI* percutaneous coronary intervention

### Effect of type of clinical presentation on outcomes

**Fig. 2:**
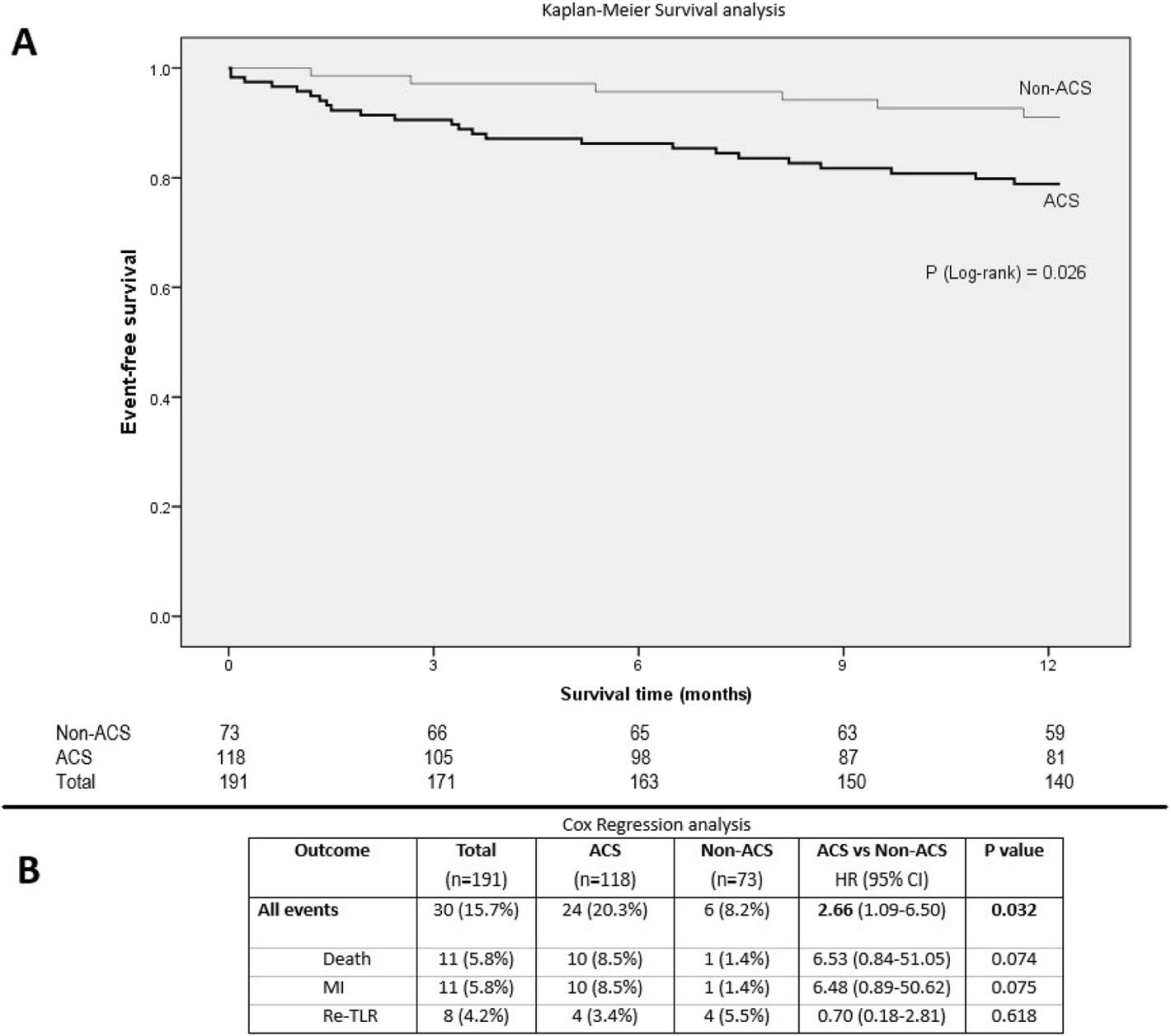
Time-to-event survival analysis at 1-year according to clinical presentation of in-stent restenosis. **a** Kaplan-Meier survival analysis. **b** Cox regression analysis. ACS: acute coronary syndrome, CI: confidence interval, HR: hazard ratio

Majority of the adverse events (24 out of 30 events, 80%) during the 1-year follow-up period occurred in patients with ISR who presented with ACS during the index hospitalization. Kaplan-Meier survival curves for ACS and non-ACS presentations are shown in Fig. 2. ISR patients presenting with ACS had a 2.66-fold higher risk of MACE at 1 year (hazard ratio [HR], 2.66; 95% CI, 1.09–6.50; *P* = 0.032). There was one case of definite stent thrombosis following PCI in the ACS group.

Within the ACS group, patients presenting with MI had a higher 1-year event rate compared to those who presented with unstable angina (24.2% and 15.4% respectively), but the difference was not statistically significant.

In the overall cohort (191 patients), there was no difference in 1-year outcomes with respect to the treatment received (event rate in medical therapy vs. CABG vs. PCI, 16.2% vs. 14.0% vs. 16.3%; *P* = 0.928).

## Discussion

Our study is the first of its kind to explore DES ISR clinical presentation and outcomes irrespective of the treatment strategy used. The main findings of this study, which included 191 patients with 210 culprit ISR lesions, were (1) ACS is the most common clinical presentation of DES ISR, (2) female gender and chronic kidney disease correlated with ACS ISR presentation and (3) ACS presentation of ISR was associated with significantly higher mortality and MACE at 1-year follow-up compared to non-ACS presentation.

### Clinical presentation of DES-ISR

Our study found that ACS (62% of patients) is the dominant presentation mode for DES-ISR and 34% of patients presented with MI. An acute unstable presentation was seen in up to 70% of patients with ISR in both BMS and DES eras of whom 10–20% presented with MI [11, 12, 19–23]. A recent study showed that ACS is the common ISR presentation mode across three stent generations but suggested that second-generation DES may present less often with MI [11]. In addition to this, the risk of late stent thrombosis with DES, albeit small, adds to this problem [24]. Despite the advantages of DES over BMS in reducing the incidence of ISR, the propensity of ISR to present with ACS has remained largely similar irrespective of stent type and has important therapeutic and prognostic implications.

### Mechanism of ACS ISR presentation

An enhanced local inflammatory reaction, fibroatheromas with thin caps, higher lipid content in plaques and/or superimposed thrombus may contribute to ACS ISR presentation with DES. Several studies have confirmed these theories by demonstrating thrombi overlying neointimal disruptions using intravascular imaging or by demonstrating fibrin/thrombi in ISR tissue of patients presenting with ACS [25, 26]. Some studies have suggested that, compared to first-generation DES, second-generation DES are associated with better vascular healing, lower prevalence of neoatherosclerosis and reduction in the incidence of unstable features such as disrupted neointima, thin-cap fibroatheroma, thrombus and fibrin deposition [27, 28]. In our setting, a plethora of stent types with various combinations of anti-proliferative drugs and polymers are available which makes it difficult to analyse the results stratified by stent types [29]. Mechanisms underlying ACS presentations with various stent types need further studies.

### Clinical correlates of DES-ISR presentation

Our study found that female gender and chronic kidney disease are significantly associated with ACS presentation. Further, patients presenting with ACS are more likely to have congestive heart failure compared to those presenting with stable syndromes. However, we did not find an association between age, body mass index, current tobacco use, history of previous MI or CABG, diabetes, hypertension or dyslipidemia and ACS presentation. Type of ISR and its location also did not correlate with presentation mode.

Women are known to present more often with atypical chest pain and angina equivalents such as dyspnea, fatigue, indigestion and weakness which may lead to delayed diagnosis and management of coronary disease [30–32]. However, these studies were conducted in women at first presentation of ischemic heart disease (IHD). Patients who present with ISR are already under treatment for IHD which makes it less likely that atypical symptoms would be ignored. This is probably the reason why none of the previous studies has found the patient’s gender to be related to the ISR presentation mode [11, 12]. Reasons for women with ISR presenting more often as ACS in our study are unclear. Women’s health receives less attention compared to their male counterparts especially in developing countries and therefore may not receive medical attention unless a more dramatic presentation ensues [33–36]. Whether such sociocultural factors contributed to our study finding needs further exploration.

Chronic kidney disease has been shown to be associated with poor outcomes after PCI with both BMS and DES era. It was found to be a factor independently associated with ISR presenting as MI [11]. This is likely explained by the increased incidence of neoatherosclerosis and higher lipid content in neointima among patients with CKD [28, 37].

Some studies found smoking to be associated with ACS ISR presentation [11]. Our study did not find an association between tobacco use and ACS presentation even after adjustment for gender differences. Differences in the way tobacco is consumed by the study population (smoking vs. chewing) and a possible reduction in the quantity of tobacco consumed due to repeated counselling during clinical visits may underlie these findings.

Although the development of ISR per se has been attributed to patient-related, stent-related and technical factors, clinical presentation mode appears to be related to patient-related factors alone [11, 12]. It is therefore important to identify patients who are at higher risk of ACS ISR presentation. Women who receive DES, and their caregivers, need to be counselled regarding regular clinical follow-up and the importance of seeking timely medical attention.

### Clinical outcomes

In our study, the clinical presentation mode affected patient outcomes. ACS ISR presentation was independently associated with a higher incidence of composite clinical outcome of death, MI and re-TLR at 1-year follow-up compared to a non-ACS presentation. Similar findings were reported by several previous studies on both BMS and DES restenosis [11, 12, 21, 22, 38, 39]. At least one previous study even reported that ISR presentation as MI may be worse than stent thrombosis [40].

ACS presentation was shown to be associated with a higher incidence of MACE and TLR in BMS ISR in the PRESTO trial [21]. Similarly, a higher risk of re-TLR was seen when patients with a first-generation DES presented with unstable angina compared to stable syndromes [38]. One study even suggested that DES ISR may be associated with poorer outcomes compared to BMS ISR for an identical level of cardiac risk [12]. Recently, a large study concluded that ACS ISR presentation is a harbinger of worse outcomes across all three stent generations (BMS, first- and second-generation DES) [11]. It is therefore pertinent to identify those at risk of ACS presentation and to closely follow ISR patients who presented with an ACS. Novel treatment strategies may be needed to improve the outcomes of patients with ACS ISR presentation.

In our entire DES-ISR cohort (191 patients), no difference in outcomes at 1-year follow-up was seen between groups receiving medical therapy, CABG and PCI. The treatment strategy was solely based on the physician’s discretion which is an important confounding factor. Therefore, this finding cannot be used to conclude that all treatment strategies are equally effective in DES ISR. We could not come across any study comparing medical therapy, CABG and PCI for DES ISR. Further research using a randomized controlled trial design are needed to compare outcomes among different treatment modalities.

### Limitations

This is a retrospective observational study, and therefore, the results may be affected by various confounding factors. The findings of this study should, therefore, be considered hypothesis-generating.

Despite the rigorous process of adjudication used, the possibility of late stent thrombosis masquerading as ISR with MI cannot be excluded. Recent studies with intravascular imaging have suggested that ISR and stent thrombosis may not be entirely distinct clinical entities.

Type of DES (first- vs. second-generation DES) received by study patients in their initial procedure (prior to the development of ISR) could not be ascertained in all patients. Therefore, the impact of the type of DES on clinical presentation could not be compared. However, in developing countries like ours, a variety of stent types with various combinations of anti-proliferative drugs and polymers are available which makes it difficult to segregate them into two or three groups for study purposes [29].

Treatment modalities could not be compared because patients were treated according to physician discretion with either PCI, CABG or medical management. Because re-TLR cannot occur in the latter two groups, re-TLR rates in our study are consequently lower. Further, the type of PCI (new DES, DCB or POBA) may also have influenced outcomes. However, we believe our study is representative of the entire spectrum of clinical ISR in the real-world situation where numerous factors affect treatment decisions and outcomes.

## Conclusions

DES-ISR presents more often as ACS, and patient-related factors like female gender and chronic kidney disease are associated with an ACS presentation. ACS presentation is independently associated with poorer clinical outcomes.

In-stent restenosis continues to be an important problem even in the current DES era because of the propensity to present as ACS. Close monitoring of patients with ACS ISR presentations is necessary. Finding and addressing the causes of gender differences in the clinical presentation of ISR may improve clinical outcomes among women. There is a need for new approaches or technologies to completely eradicate the problem of ISR.

## Data Availability

The dataset supporting the results and conclusions of this article will be available from the corresponding author on request.

## Abbreviations

ACS: Acute coronary syndrome
ASCVD: Atherosclerotic cardiovascular disease
BMS: Bare-metal stent
CABG: Coronary-artery bypass grafting
CCF: Congestive cardiac failure
DES: Drug-eluting stent
ISR: In-stent restenosis
MACE: Major adverse cardiac events
MI: Myocardial infarction
PCI: Percutaneous coronary intervention
TLR: Target lesion revascularization
